# lncRNA 00312 Attenuates Cell Proliferation and Invasion and Promotes Apoptosis in Renal Cell Carcinoma via miR-34a-5p/ASS1 Axis

**DOI:** 10.1155/2020/5737289

**Published:** 2020-03-23

**Authors:** Jiawei Zeng, Yuanmeng Li, Yaodong Wang, Gang Xie, Qian Feng, Yuwei Yang, Jiafu Feng

**Affiliations:** ^1^Department of Clinical Laboratory, Mianyang Central Hospital, Affiliated to Southwest Medical University, Mianyang 621000, Sichuan Province, China; ^2^Department of Medical Laboratory, Affiliated Hospital of Southwest Medical University, Luzhou 646000, Sichuan Province, China; ^3^Urology Surgery, Mianyang Central Hospital, Affiliated to Southwest Medical University, Mianyang 621000, Sichuan Province, China; ^4^Pathology, Mianyang Central Hospital, Affiliated to Southwest Medical University, Mianyang 621000, Sichuan Province, China; ^5^College of Medical Technology, Chengdu University of Traditional Chinese Medicine, Chengdu 610075, Sichuan Province, China

## Abstract

**Background:**

Previous studies have demonstrated that lncRNAs play functional roles in regulating cancer cell proliferation, invasion, and apoptosis. Recent studies confirmed that lncRNA 00312 has important biological functions in lung and colorectal cancer. However, the role of lncRNA 00312 in renal cell carcinoma (RCC) remains unclear. Our aim was to explore the function of lncRNA 00312 in RCC and its potential molecular mechanism.

**Methods:**

RCC cell lines A498 and ACHN were used as *in vitro* models in this study. RT-PCR was performed to determine lncRNA 00312, miR-34a-5p, and ASS1 mRNA expression. Proliferation and invasion were examined by CCK-8 and Transwell assay to confirm the function role of lncRNA 00312. Western blot analysis was used to examine the expression of apoptotic proteins Bax and Bcl-2.

**Results:**

lncRNA was significantly downregulated in RCC cells such as A498 and ACHN; the expression of lncRNA 00312 in RCC tissues was significantly lower than that in adjacent normal tissues. Patients with low expression of lncRNA 00312 have worse prognosis regarding pathological grade, tumor size, and TNM stage. Overexpression of lncRNA 00312 suppressed A498 and ACHN cell proliferation and invasion, while promoting apoptosis. Our study found that miR-34a-5p had the potential binding site with lncRNA 00312 and revealed the role of miR-34a-5p in RCC. Furthermore, we confirmed that lncRNA 00312 played its role with the participation of ASS1 and miR-34a-5p.

**Conclusion:**

lncRNA 00312 can inhibit RCC proliferation and invasion and promote apoptosis *in vitro* by suppressing miR-34a-5p and overexpressing ASS1. Our study demonstrated that the lncRNA 00312/miR-34a-5p/ASS1 axis may play a functional role in the progression of RCC; lncRNA 00312 abundance is a prognostic factor candidate for RCC survival, which provides new insights for RCC clinical treatment.*in vitro* models in this study. RT-PCR was performed to determine lncRNA 00312, miR-34a-5p, and ASS1 mRNA expression. Proliferation and invasion were examined by CCK-8 and Transwell assay to confirm the function role of lncRNA 00312. Western blot analysis was used to examine the expression of apoptotic proteins Bax and Bcl-2.

## 1. Introduction

Renal cell carcinoma (RCC), accounting for 90% of all renal tumors, is the most common malignant disease in the kidney [1, 2]. In addition to the large and growing number of patients with RCC, RCC is a high-mortality cancer with high malignancy and concealment. About 20% of patients are initially diagnosed at an advanced stage due to the lack of significant biomarkers, and nearly 30% of patients with RCC cannot receive surgery [3]. Currently, medication is another first-line treatment for RCC patients besides surgery [4]. However, drug resistance is a serious problem for patients with RCC causing high recurrence and metastasis rate [5]. Moreover, the molecular mechanism of the occurrence and progression of RCC is not fully known. Therefore, it is particularly important to elucidate the molecular mechanism of the occurrence and progression of RCC and identify new therapeutic targets and molecular markers for early diagnosis.

In recent years, with the wide application of high throughput sequencing technology, long-chain noncoding RNA (lncRNA), originally thought to be the “noise” of genomic transcription, has been proved to play an important biological role in many diseases [6–8]. lncRNAs are a class of transcripts with a length of more than 200 nucleotides, which cannot encode proteins or polypeptides [9]. Recent studies have shown that lncRNAs can regulate gene expression by multitarget, including participation in chromatin imprinting, binding with epigenetic modification complexes or transcription factors, and binding with microRNAs, mRNA, or proteins to perform biological functions [10–12]. Numerous evidences have shown that lncRNAs are involved in regulating various biological characteristics of tumors, including proliferation, apoptosis, metastasis, and metabolism [13, 14]. Previous microarray results showed that a large number of lncRNAs were significantly downregulated or upregulated in different cancer tissues [15, 16]. lncRNAs are also involved in the occurrence and development of RCC, such as knockdown of lncRNA ROR inhibiting the proliferation, migration, and invasion of RCC cells. ROR-induced cell proliferation and metastasis can be blocked by the overexpression of miR-206 [17]. lncRNA HOTTIP and NABT1 could regulate RCC cell proliferation and invasion and affect RCC progression [18, 19]. However, the role and mechanism of lncRNAs in RCC are not fully understood.

lncRNA 00312 (Gene ID: 29931) attracted our attention based on its highly conserved characteristics and biological function. Previous studies have confirmed that lncRNA 00312 was significantly downregulated in both colorectal and lung cancers [20, 21]. Overexpression of lncRNA 00312 can significantly inhibit the invasion and proliferation of lung cancer and colorectal cancer cells. These results demonstrate that lncRNA 00312 is a lncRNA that may have biological functions and therapeutic effects in multisystem tumors. However, there are few research findings which revealed the relationship between lncRNA 00312 and RCC.

In our study, we studied the expression of lncRNA 00312 in RCC cells and analyzed the relationship between lncRNA 00312 and clinical characteristics of RCC patients. Moreover, we will further explore the molecular mechanism and biological role of lncRNA 00312 and provide new targets for the prevention and treatment of RCC.

## 2. Materials and Methods

### 2.1. Patient and Clinical Information

A total of 47 RCC patients at Mianyang Central Hospital from January, 2017, to March, 2019, were enrolled into this study. There are 30 men and 17 women with the mean age of 56.2 ± 12.8 years (range: 36-75 years). The baseline demographic and clinical characteristics were recorded from patient interviews and chart review. Urine and blood samples were taken the morning before the operation. Urine albumin-creatinine ratio (ACR) was calculated by urinary albumin and creatinine (Cr) concentration. GFR was calculated with serum Cr and cystatin C (CysC) levels, using the CKD-EPI equation [22]. The results are shown in Table 1.

The tissue and normal tissue samples were collected from 47 RCC patients who underwent RCC surgery at the Mianyang Central Hospital. All the samples were immediately frozen in liquid nitrogen and stored at −80°C for further experiment. All excised tissues have been examined by pathologists. And none of the patients received anticancer therapy before surgery. All samples were staged according to the tumor node metastasis (TNM) classification and criteria of the World Health Organization (WHO), and tumor grade was assessed in accordance with the WHO criteria. This study was approved by the Human Ethics Committees Review Board at the Mianyang Central Hospital (S201400048, S2018085).

### 2.2. Cell Lines and Culture

RCC cell lines used in the study were all purchased from the American Type Culture Collection (ATCC) and cultured in a humidified incubator at 37°C with an atmosphere of 5% CO_2_. A498 and ACHN were cultured in MEM medium (Gibco) supplemented with 10% fetal bovine serum (Gibco), and HK2, 786-O, and 769-P were cultured in RPMI 1640 medium (Gibco) containing 10% fetal bovine serum (Gibco).

### 2.3. Cell Transfection

For overexpressing lncRNA 00312, the complementary DNA encoding lncRNA 00312 (Gene ID: 29931) was amplified by PCR from human genomic DNA (forward primer 5′-CCCAAGCTTTTTTCTTTTGATAGAATGTCAAACTTACG-3′ and reverse primer 5′-CGCGGATCCTTCAGGGAATATCATTTTATTTAGGCC-3′, adding *HindIII* and *BamHI* restriction enzyme cutting site) and then cloned into eukaryotic expression vector pcDNA3.1 (Invitrogen), forming pcDNA-lncRNA 00312. For downregulated lncRNA 00312, siRNA-mediated knockdown of lncRNA 00312 was obtained from Shanghai Genechem Co., Ltd. (China) with the following sequence: forward 5′-GGCUGUUGGUCAUUCACAUTT-3′ and reverse 5′-AUGUGAAUGACCAACAGCCTT-3′. lncRNA 00312 expression was tested by qRT-PCR assay.

MiR-34a-5p inhibitor, miR-34a-5p mimic, and ASS1 siRNA versus their respective control were obtained from Shanghai Genechem Co., Ltd. The sequence of miR-34a-5p inhibitor was 5′-CUACCUGCACCAACAGCACUU-3′, the sequence of miR-34a-5p mimic was 5′-GAUGGACGUGCUUGUCGUGAAAC-3′, and the sequence of ASS1 siRNA was 5′-AGCAGC UGAGCUCAAACCGGACCU-3′.

lncRNA 00312 overexpression plasmid, lncRNA 00312 siRNA (200 nM), miR-34a-5p mimic (50 nM), miR-34a-5p inhibitor (100 nM), and ASS1 siRNA (100 nM) were transfected into A498 and ACHN cells using Lipo3000, respectively or jointly. At 48 h posttransfection, transfected cells were harvested for the next analysis.

### 2.4. Cell Counting Kit-8 (CCK-8) Assay

RCC cell proliferation was detected using Cell Counting Kit-8 (CCK-8; Dojindo), according to the manufacturer's instructions. RCC cells were cultured in a 96-well plate and then exposed to further treatment. Approximately 10 *μ*L of CCK8 reagent was added per well and then incubated at 37°C for 2 hours. Cell growth was analyzed at 0, 12, 24, and 48 h after transfection. The absorbance at 490 nm was detected by using a microplate reader (Thermo Fisher Scientific).

### 2.5. Real-Time Polymerase Chain Reaction (qRT-PCR) Analysis

Sample RNAs were obtained from whole cell lysate or specific subcellular fractions using PARIS™ Kit (Applied Biosystem). cDNA was converted from RNA by using a Reverse Transcription Kit (Takara) and performed for further quantitative real-time PCR with SYBR Green (Applied Biosystem) according to the manufacturer's instructions. Specific gene measurement was performed by qRT-PCR analysis according to the manufacturer's instructions. The procedure of qRT-PCR was as follows: 95°C for 2 min, followed by 45 cycles at 95°C for 15 s and 60°C for 45 s. The relative level of related gene expression was compared with the respective internal control using 2^-*ΔΔ*Ct^ methods. The primers used in the qRT-PCR are shown in Table 2. GAPDH was used as a normalized control.

### 2.6. Western Blotting Analysis

Western blots were performed using standard procedures. Briefly, cell lysates were prepared using RIPA buffer. Equivalent proteins were separated by SDS-PAGE and transferred onto nitrocellulose blotting membranes (Millipore). Subsequently, the membranes were blocked with 5% BSA for 2 h and then incubated with specific primary antibodies GAPDH (Santa Cruz Biotechnology, CA, USA, 1 : 1000), Bax (Santa Cruz Biotechnology, CA, USA, 1 : 1000), and Bcl-2 (Santa Cruz Biotechnology, CA, USA, 1 : 1000) overnight at 4°C. GAPDH was used as a normalized control. And then membranes were incubated with secondary antibody (Santa Cruz Biotechnology, CA, USA, 1 : 1000) at room temperature for 2 h. The protein bands were finally detected using an Odyssey infrared scanner (Li-Cor Biosciences Inc.) and analyzed using ImageJ software after being normalized to its internal control.

### 2.7. Luciferase Reporter Assay

Software starBase V2.0 was used to predict the theoretical binding sequence of lncRNA 00312 and miR-34a-5p.The 250 bp sequence including the theoretical binding site with miR-34a-5p in the lncRNA 00312 was obtained via PCR amplification (forward primer 5′-GCTCTAGACAGACCTCCCGAAGGCTT-3′ and reverse primer 5′-ATAAGAATGCGGCCGGCTTATTCTGGCCAAACCCAC-3′) and cloned into the pGL3-Basic Vector (Promega, Madison, WI, USA) to construct a dual luciferase reporter plasmid (wt-luc vector). MutaBest kit (Takara, Tokyo, Japan) was then used to generate lncRNA 00312 mutant-luc vector. A498 and ACHN cells were transfected with wt-luc vector (or mutant-luc vector) reporter plasmid and a negative control mimic or miR-34a-5p mimic. Forty-eight hours after transfection, luciferase activities were examined by using a dual luciferase reporter assay system (Promega).

### 2.8. Transwell Assay

The invasion of RCC cells was calculated based on the number of transfected cells through Transwell inserts (8-micron chamber; Corning Life Sciences). Firstly, Transwell inserts were coated with 100 *μ*L Matrigel (BD Biosciences, San Jose) at 37°C for 5 h until gelling. Then 1 × 10^5^ A498 and ACHN cells, which were starved for 24 h, were seeded into the upper chamber in a 500 *μ*L serum-free MEM medium, respectively (each group consisted of three technical replicates). The lower chamber added 700 *μ*L MEM medium containing 10% fetal bovine serum. After plate incubation for 48 h at 37°C, Matrigel and noninvasive cells were removed from the upper surface of filters. Cells adhering to the under surface of filters were fixed with 4% paraformaldehyde and stained with crystal violet. They were then counted and imaged with a Nikon digital camera (magnification ×100). The invasion assay was repeated in three independent experiments.

### 2.9. Apoptosis Assay

RCC cell apoptosis was examined using terminal deoxynucleotidyl transferase-mediated dUTP nick-end-labeling assay (TUNEL) kit (Roche) according to the manufacturer's instruction. After TUNEL staining, images were captured by a Fluorescence Microscope System (Carl Zeiss).

### 2.10. Statistical Analysis

All data were analyzed for statistical significance using GraphPad Prism software 6.0. All results were presented as the means ± SD. An independent samples *t*-test was used for two-group comparison and one-way ANOVA for multiple-group comparisons followed by Bonferroni's post hoc test. *P* < 0.05 was considered statistically significant.

## 3. Results

### 3.1. Expression Features of lncRNA 00312

**Figure 1:**
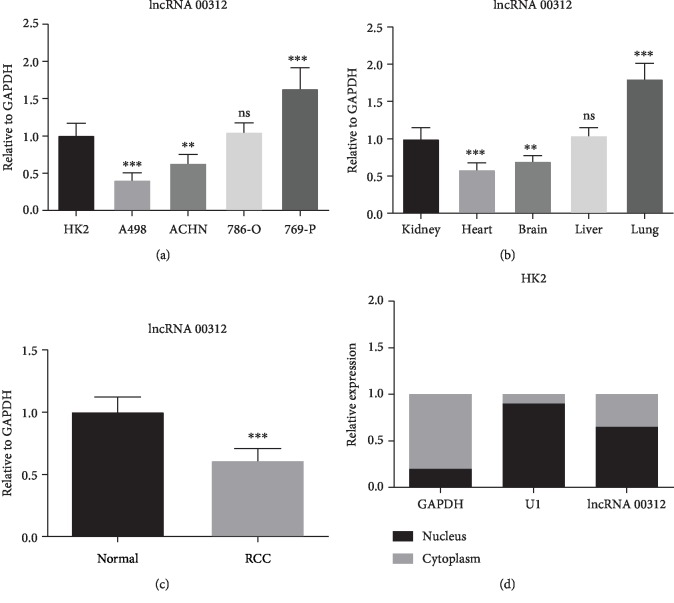
Biological features of lncRNA 00312. (a) Relative expression of lncRNA 00312 in different RCC cell lines (*n* = 6). HK2: human HK2 renal proximal tubular epithelial cells on half of normal renal epithelial cells; A498: human renal epithelial cell carcinoma on half of RCC cells; ACHN: human renal adenocarcinoma cell on half of RCC cells; 786-O: human clear cell RCC on half of RCC cells; 769-P: human renal adenocarcinoma cell on half of RCC cells; ns: not significant. (b) The expressive abundance of lncRNA 00312 in different tissues of mice was detected via RT-PCR analysis (*n* = 6). ns: not significant. (c) The expression of lncRNA 00312 was decreased in RCC tissues compared to normal tissue (*n* = 6). (d) Location of lncRNA 00312 was examined via extracting specific subcellular fractions from HK2 cell line (*n* = 6). ^∗∗^*P* < 0.01 and ^∗∗∗^*P* < 0.001 versus respective control; ns: not significant.

To confirm the expression characteristics of lncRNA 00312, we firstly examined and compared the lncRNA 00312 expression in RCC cell lines (A498, ACHN, 786-O, and 769-P) and normal renal cell line (HK2). The qRT-PCR results showed that lncRNA was markedly downregulated in A498 and ACHN cells while being upregulated in 769-P cells compared to HK2 (Figure 1(a)). So A498 and ACHN cells were chosen to artificially overexpress lncRNA00312 *in vitro*. Then, we determined the lncRNA 00312 expression in tissues of different organs. We found that lncRNA 00312 expression decreased in the brain and heart while it increased in the lung compared with kidney tissues (Figure 1(b)). In addition, we confirmed that the expression of lncRNA 00312 in RCC tissues was significantly lower than that in adjacent normal tissues (Figure 1(c)). Finally, we carried out a nuclear-cytoplasmic separation experiment and confirmed that lncRNA 00312 is partially or mainly expressed in the nucleus (Figure 1(d)).

### 3.2. lncRNA 00312 Expression Is Associated with RCC Patients' Clinical Progression

To explore the clinical significance of lncRNA 00312 in RCC patients, 47 RCC tissue samples were extracted and examined for their mRNA levels. Then, the association between lncRNA 00312 expression and pathological features of RCC was examined. The RNA samples were assigned into the “low-expression” (*n* = 22) and “high-expression” (*n* = 25) lncRNA 00312 groups based on the median value of lncRNA 00312 expression (1.4 ± 0.1). As shown in Table 1, low expression of lncRNA 00312 was significantly associated with pathological grade (*P* = 0.024), tumor size (*P* = 0.030), and TNM stage (*P* = 0.028). These data suggest that lncRNA 00312 expression is closely related to clinical progression of RCC.

### 3.3. lncRNA 00312 Inhibits Invasion and Proliferation while Enhancing Apoptosis in RCC Cells

**Figure 2:**
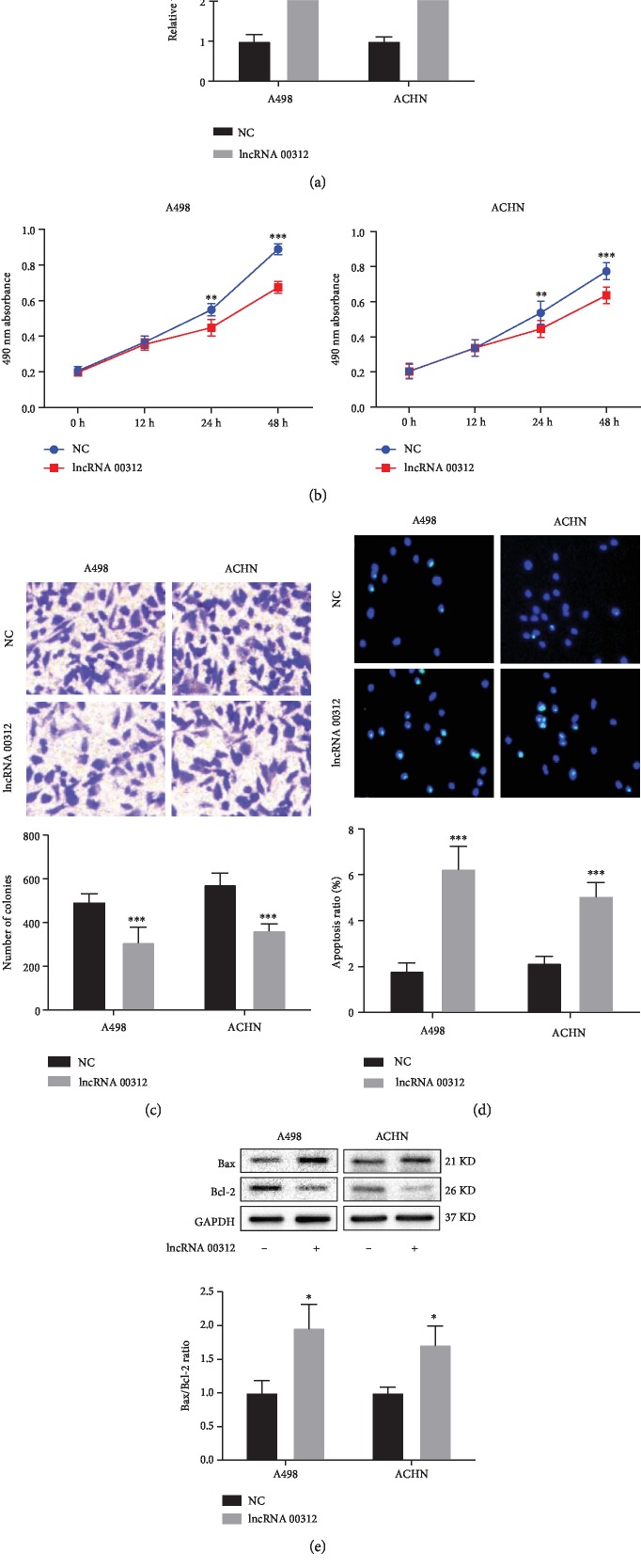
Function of lncRNA 00312 was detected in RCC cell lines after overexpression. (a) Efficacy of overexpressing lncRNA 00312 was verified via real-time PCR (*n* = 6). NC: cells transfected empty vector as negative control. (b) lncRNA 00312 inhibited cell proliferation in A498 and ACHN cell lines (*n* = 6). NC: cells transfected empty vector as negative control. (c) lncRNA 00312 inhibited cell invasion in A498 and ACHN cell lines (*n* = 6). NC, cells transfected empty vector as negative control. (d, e) lncRNA 00312 promoted cell apoptosis indicated by TUNEL staining (*n* = 6) and Western blot analysis (*n* = 3). NC: cells transfected empty vector as negative control. ^∗^*P* < 0.05, ^∗∗^*P* < 0.01, and ^∗∗∗^*P* < 0.001 versus the respective control.

To study the function of lncRNA 00312 in RCC, we used lncRNA 00312 overexpression vector to upregulate the expression of lncRNA 00312 in A498 and ACHN cells, respectively (Figure 2(a)). CCK-8 assay results demonstrated that overexpression of lncRNA 00312 significantly suppresses the proliferation in two RCC cell lines (Figure 2(b)). In addition, Transwell assay results confirmed that lncRNA 00312 overexpression significantly attenuates the cell invasion in A498 and ACHN cells (Figure 2(c)). Lastly, TUNEL staining results clarified that lncRNA 00312 overexpression obviously promoted the apoptosis rate in A498 and ACHN cells (Figure 2(d)). Western blot analysis results also showed overexpression of lncRNA 00312 reduced the protein expression of Bcl-2, whereas it increased the Bax expression and Bax/Bcl-2 ratio in A498 and ACHN cells (Figure 2(e)).

### 3.4. Knockdown of lncRNA 00312 Promotes Invasion and Proliferation while Inhibiting Apoptosis in RCC Cells

**Figure 3:**
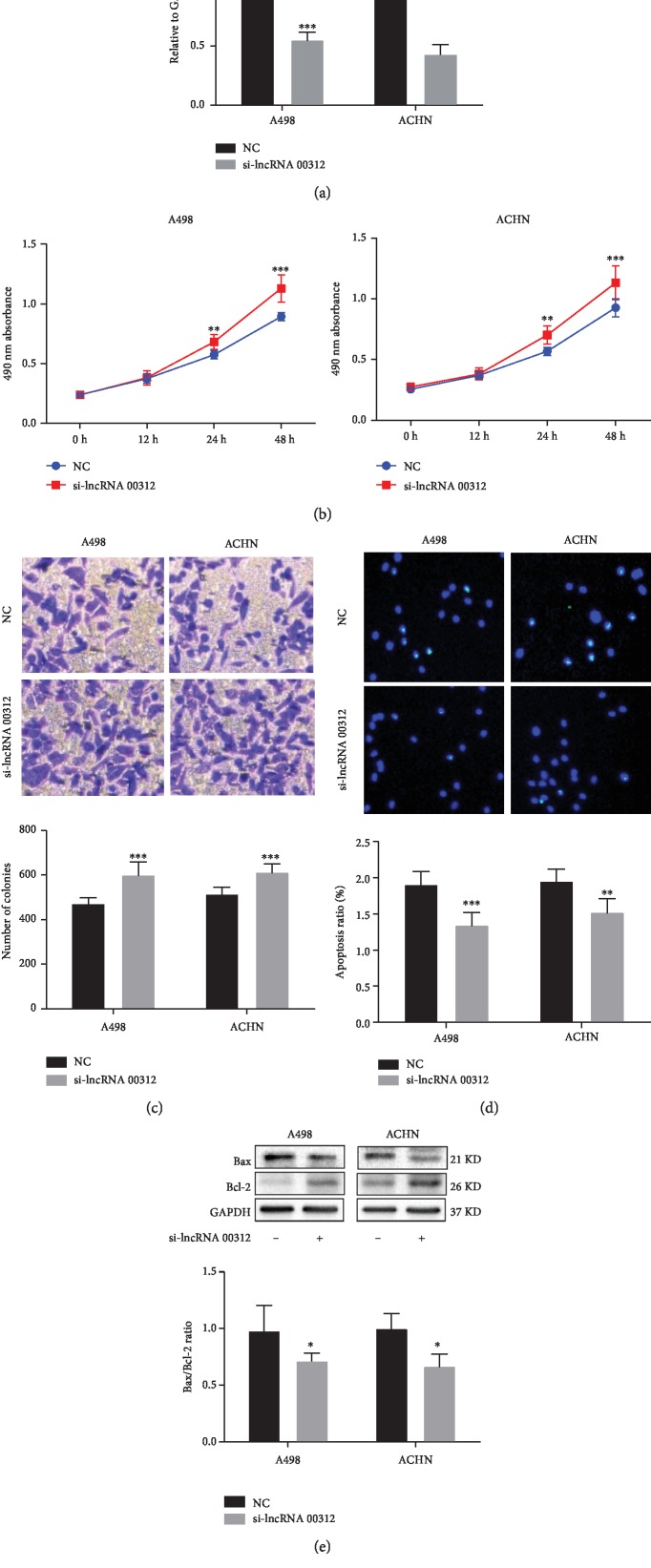
Function of RCC cell lines was detected after inhibiting lncRNA 00312. (a) Inhibition of lncRNA 00312 was verified via real-time PCR (*n* = 6). NC: cells transfected empty vector as negative control. (b) Inhibition of lncRNA 00312 increased cell proliferation in A498 and ACHN cell lines (*n* = 6). NC: cells transfected empty vector as negative control. (c) Inhibition lncRNA 00312 promoted cell invasion in A498 and ACHN cell lines (*n* = 6). NC: cells transfected empty vector as negative control. (d, e) Cell apoptosis was decreased after inhibiting lncRNA 00312 indicated by TUNEL staining (*n* = 6) and Western blot analysis (*n* = 3). NC: cells transfected empty vector as negative control. ^∗^*P* < 0.05, ^∗∗^*P* < 0.01, and ^∗∗∗^*P* < 0.001 versus respective control.

To further confirm the function role of lncRNA 00312 in RCC, we constructed a knockdown vector to inhibit lncRNA 00312 expression verified by qRT-PCR (Figure 3(a)). Inhibition of lncRNA 00312 significantly enhanced the cell proliferation in A498 and ACHN cells using CCK-8 assay (Figure 3(b)). The number of invasion cells was also increased after the inhibition of lncRNA 00312 in A498 and ACHN cells (Figure 3(c)). In addition, TUNEL assay and Western blot analysis results showed that inhibition of lncRNA 00312 significantly suppresses the apoptosis in A498 and ACHN cells (Figures 3(d) and 3(e)).

### 3.5. lncRNA 00312 Exerts Biological Functions in Renal Cell Carcinoma through Regulating miR-34a-5p

**Figure 4:**
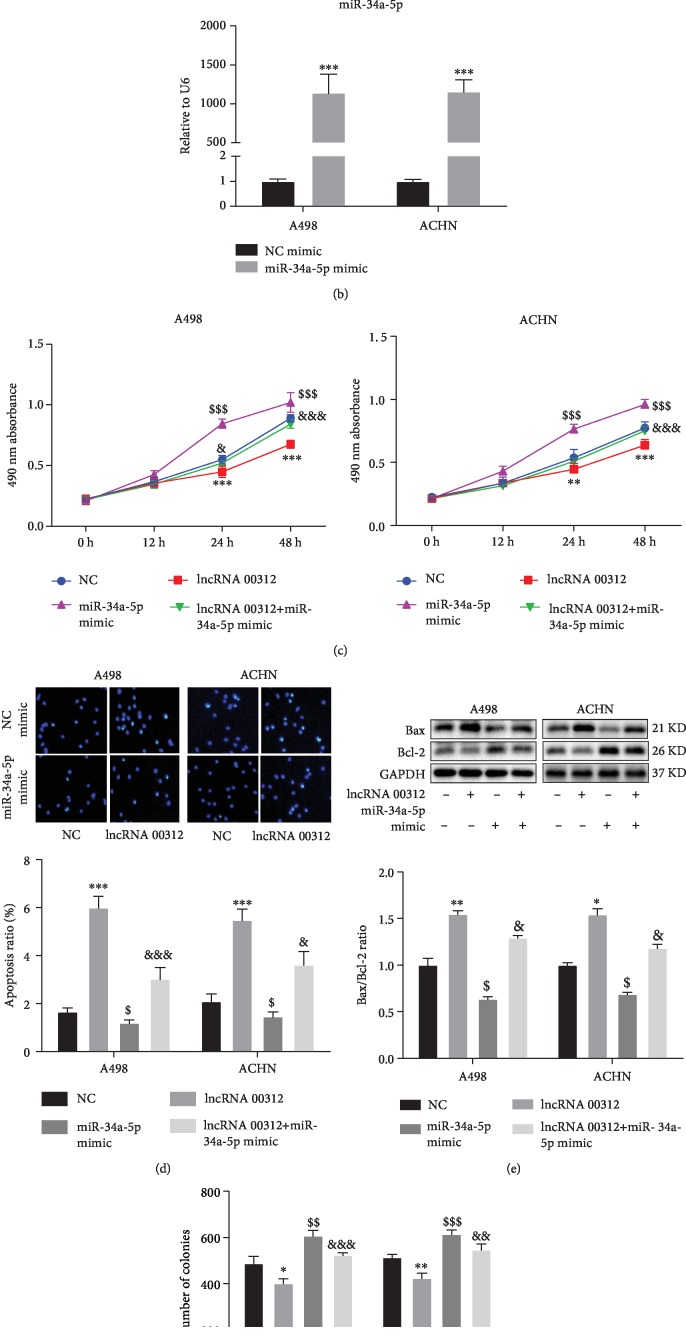
miR-34a-5p negatively regulates the function of lncRNA 00312. (a) Luciferase reporter assay indicated miR-34a-5p is the target of lncRNA 00312. (b) Efficacy of miR-34a-5p mimic was detected via RT-PCR analysis (*n* = 6). (c) miR-34a-5p mimic promoted proliferation and inhibited function of lncRNA 00312 in A498 and ACHN cell lines (*n* = 6). NC mimic: cells transfected unrelated RNA as negative control; NC inhibitor: cells transfected empty vector of miR-34a-5p inhibitor as negative control. (d, e) miR-34a-5p mimic attenuated effect on promoting apoptosis of lncRNA 00312 and further inhibited cell apoptosis by TUNEL staining (*n* = 6) and western blot analysis (*n* = 3). (f) Invasion of RCC cells was determined after lncRNA 00312 and transfection of miR-34a-5p mimic (*n* = 6). NC: cells transfected nonsense sequence and empty vector as negative control. ^∗,$,&^*P* < 0.05, ^∗∗,$$,&&^*P* < 0.01, and ^∗∗∗,$$$,&&&^*P* < 0.001 versus respective control (multiple comparison: ^∗^NC group versus lncRNA 00312 group; ^$^NC group versus miR-34a-5p mimic group; ^&^lncRNA 00312 group versus lncRNA+miR-34a-5p mimic group).

Software analysis (starBase, V2.0) was used to predict the potential target of lncRNA 00312. As shown in Figure 4(a), miR-34a-5p was predicted as the possible target of lncRNA 00312. Then, dual-luciferase reporter assay was conducted to verify whether miR-34a-5p was the target of lncRNA 00312. The results indicated that inhibition of miR-34a-5p could increase the relative luciferase activity of lncRNA 00312 vector while overexpression of miR-34a-5p could reduce the relative luciferase activity of lncRNA 00312 in the WT 3′ group. Nevertheless, no significant difference was examined in the mutant 3′ group. In order to confirm miR-34a-5p was the target of lncRNA 00312, we transfected RCC cells with lncRNA 00312 overexpression vector and/or miR-34a-5p mimic, to explore whether miR-34a-5p was involved in the function of lncRNA 00312. Efficiency of miR-34a-5p mimic was detected by qRT-PCR analysis (Figure 4(b)). The cells transfected with miR-34a-5p mimic had more than 1100 times miR-34a-5p compared with negative control. Then, we explored the association between miR-34a-5p and lncRNA 00312. We found that miR-34a-5p mimic can restore the effect of lncRNA 00312 on proliferation of A498 and ACHN cell lines (Figure 4(c)). TUNEL assay and Western blot analysis results confirmed that miR-34a-5p mimic can partially block the role of lncRNA 00312 on apoptosis in two RCC cells (Figures 4(d) and 4(e)). Lastly, we also demonstrated that the overexpression role of lncRNA 00312 mimic on cell invasion in two RCC cells was reversed by miR-34a-5p mimic treatment (Figure 4(f)).

### 3.6. The Role of lncRNA 00312 Inhibition in RCC Cells Can Be Reversed by miR-34a-5p Overexpression

**Figure 5:**
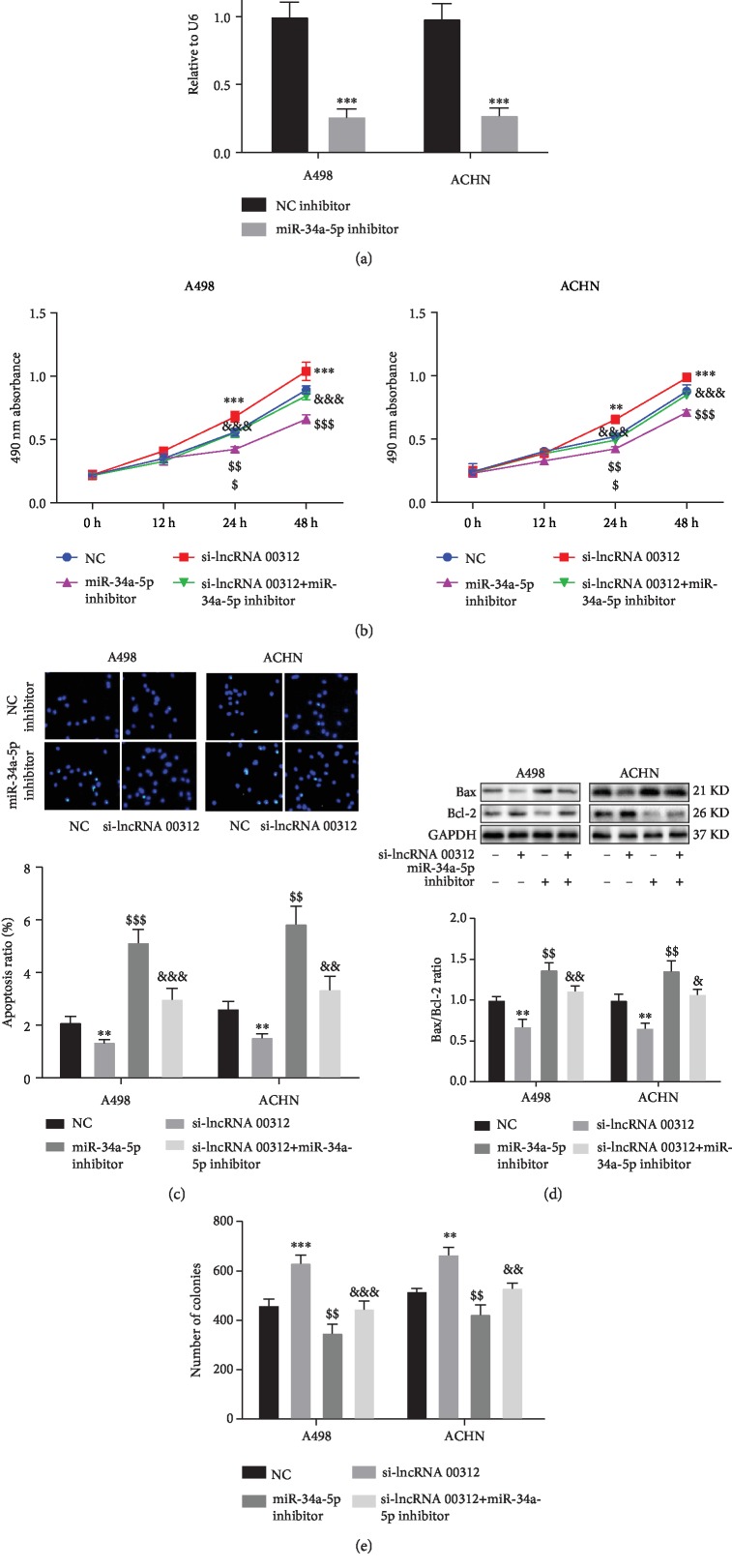
miR-34a-5p inhibitor diminished function of inhibiting lncRNA 00312. (a) qRT-PCR analysis revealed efficacy of miR-34a-5p inhibitor (*n* = 6). NC inhibitor: cells transfected empty vector as negative control. (b) miR-34a-5p inhibitor blocks lncRNA 00312 siRNA pro-proliferation in RCC cell lines (*n* = 6). NC: cells transfected empty vector as negative control. (c, d) TUNEL staining (*n* = 6) and Western blot analysis (*n* = 3) exhibited miR-34a-5p inhibitor which can partially block the inhibition of lncRNA 00312 siRNA on apoptosis in two RCC cells. NC: cells transfected empty vector as negative control. (e) miR-34a-5p inhibitor could reverse the invasive effect of inhibiting lncRNA 00312 (*n* = 6). NC: cells transfected empty vector as negative control. ^∗,$,&^*P* < 0.05, ^∗∗,$$,&&^*P* < 0.01, and ^∗∗∗,$$$,&&&^*P* < 0.001 versus respective control (multiple comparison: ^∗^NC group versus si-lncRNA 00312 group; ^$^NC group versus miR-34a-5p inhibitor group; ^&^si-lncRNA 00312 group versus si-lncRNA 00312+miR-34a-5p inhibitor group).

To further elucidate the relationship between lncRNA 00312 and miR-34a-5p in RCC, we used lncRNA 00312 siRNA and/or miR-34a-5p inhibitor to transfect RCC cells, respectively or jointly. The expression level of miR-34a-5p is shown in Figure 5(a). The A498 and ACHN cells transfected with miR-34a-5p inhibitor had only approximately 30% miR-34a-5p compared with negative control. CCK-8 assay results showed that the RCC cell proliferation was elevated by lncRNA 00312 inhibition, while was blocked after miR-34a-5p inhibitor treatment (Figure 5(b)). TUNEL assay and Western blot analysis results indicated that miR-34a-5p inhibitor could partially blocked the suppressive role of lncRNA 00312 siRNA on apoptosis in two RCC cells (Figures 5(c) and 5(d)). Similar to the above results, Transwell assay demonstrated that the promotion role of lncRNA 00312 siRNA on cell invasion in two RCC cell lines was blocked by miR-34a-5p inhibitor treatment (Figure 5(e)).

### 3.7. lncRNA 00312 Exerts Biological Roles via ASS1 in Renal Cell Carcinoma

**Figure 6:**
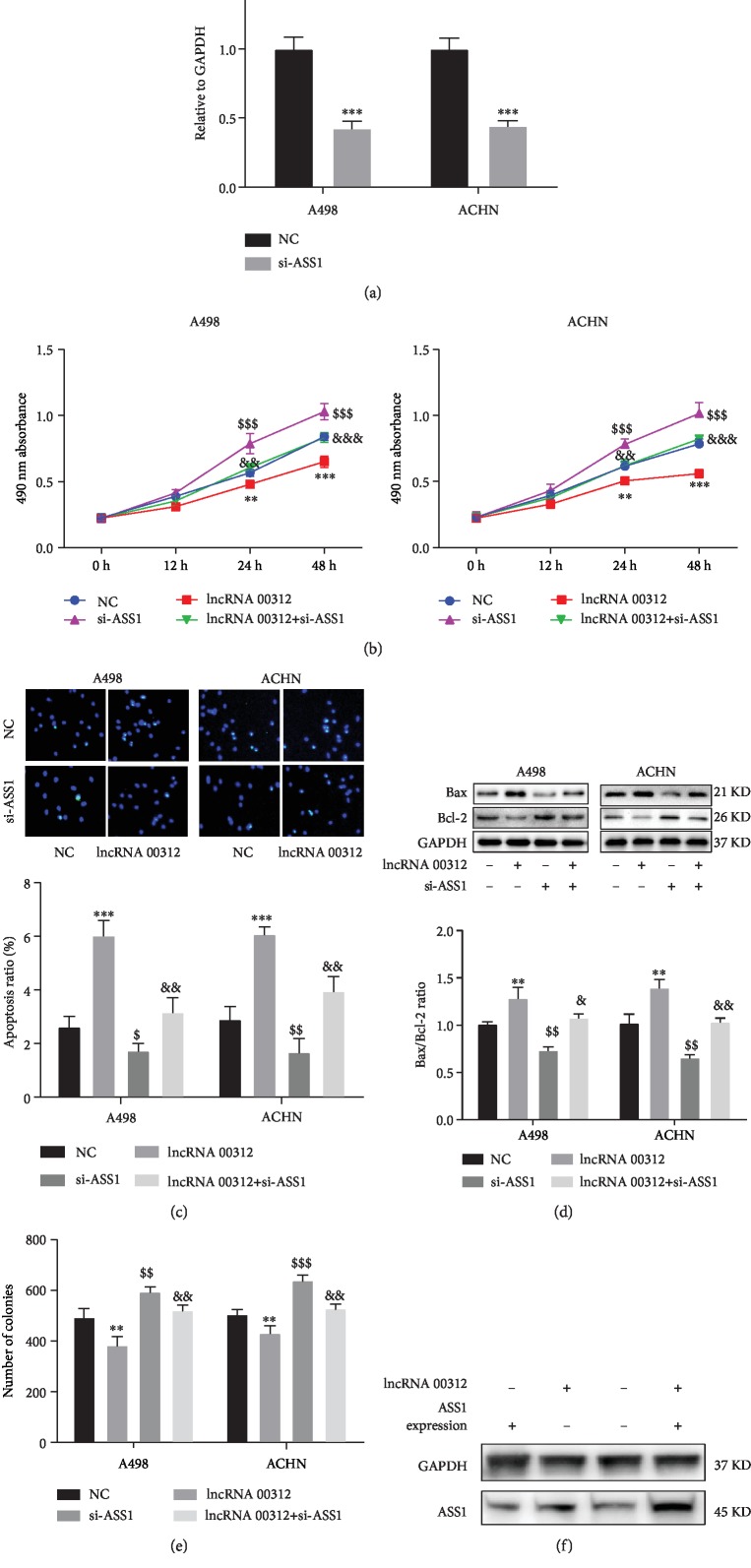
lncRNA 00312 performed its function via ASS1. (a) RT-PCR analysis determined efficacy of interfering ASS1 (*n* = 6). NC: cells transfected two empty vectors as negative control. (b) Interference of ASS1 could increase cell proliferation and inhibited function of lncRNA 00312 (*n* = 6). NC: cells transfected two empty vectors as negative control. (c, d) Inhibiting ASS1 diminished the effect on promoting apoptosis of lncRNA 00312 and further inhibited cell apoptosis by TUNEL staining (*n* = 6) and Western blot analysis (*n* = 3). NC: cells transfected two empty vectors as negative control. (e) ASS1 inhibition increased cell invasion and attenuated the effect of lncRNA 00312 inhibiting cell invasion (*n* = 6). ^∗,$,&^*P* < 0.05, ^∗∗,$$,&&^*P* < 0.01, and ^∗∗∗,$$$,&&&^*P* < 0.001 versus respective control (multiple comparison: ^∗^NC group versus lncRNA 00312 group; ^$^NC group versus si-ASS1 group; ^&^lncRNA 00312 group versus lncRNA 00312+si-ASS1 group). NC: cells transfected nonsense sequence as negative control. (f) Western blot analysis result for ASS1 expression. lncRNA 00312+: cells transfected lncRNA 00312 overexpression vector; lncRNA 00312-: cells transfected empty vector of lncRNA 00312 overexpression vector; ASS1 expression +: cells transfected ASS1 overexpression plasmid; ASS1 expression -: cells transfected empty si-ASS1 overexpression plasmid.

Previous studies have shown that miR-34a-5p can bind and negatively regulate ASS1 expression to exert biological functions [23]. In this study, in order to verify whether ASS1 is involved in the function of lncRNA 00312 in RCC, we transfected si-ASS1 and lncRNA 00312 overexpression vector in RCC cells. The siRNA successfully inhibited ASS1 expression in A498 and ACHN cells (Figure 6(a)). The Western blot result showed that overexpression of lncRNA 00312 could increase the ASS1 content directly (Figure 6(f)). The CCK-8 assay results indicated that the proliferation of RCC cells inhibited by lncRNA 00312 overexpression was blocked after ASS1 inhibition (Figure 6(b)). TUNEL assay and Western blot analysis results confirmed that inhibition of ASS1 can partially reversed the promotion of apoptosis by lncRNA 00312 in two RCC cells (Figures 6(c) and 6(d)). Transwell assay results demonstrated that inhibition of invasion by lncRNA 00312 overexpression in two RCC cell lines was blocked by ASS1 siRNA treatment (Figure 6(e)).

## 4. Discussion

At present, renal cell carcinoma is one of the most malignant cancers due to lack of effective clinical treatment and diagnostic biomarkers [24]. It is urgent and important to explore the biological mechanism of RCC and find new therapeutic targets to prevent metastasis. Numerous studies have confirmed that lncRNAs play an important role in many biological processes, such as development, cell growth, and tumorigenesis [4, 25]. lncRNA 00312 is one of them; former studies have proved that it had the potential to be the biomarker and new drug for liver cancer [16], lung cancer [20], and colorectal cancer [21].

In our study, we confirmed that lncRNA 00312 expression was significantly downregulated in RCC tissues. We not only investigated the role of lncRNA 00312 in RCC cells but also analyzed the relationship between lncRNA 00312 and clinical characteristics of RCC according to the expression level of lncRNA 00312 in 47 RCC cases. The results showed that the lower expression of lncRNA 00312 in RCC was a worse prognostic factor regarding the tumor size, pathological grade, and TNM stage of those RCC patients. These results suggest that lncRNA 00312 abundance was a prognostic factor candidate for RCC survival.

By overexpressing lncRNA 00312 in RCC cells, we revealed that lncRNA 00312 can inhibit the proliferation, invasion, and apoptosis of RCC cells. These results suggest that lncRNA 00312 may be a potential therapeutic gene in RCC patients.

After defining the function of lncRNA 00312 in RCC, we further studied how lncRNA 00312 regulates the invasion, proliferation, and apoptosis of RCC cells. Since lncRNA 00312 is mainly located in the cytoplasm of RCC cells, it may act as an endogenous competitive RNA (ceRNA) to bind to microRNAs, thus eliminating the inhibition of microRNAs on target gene transcripts. Using a bioinformatics database (starBase), we predicted that there were binding sites of miR-34a-5p in the 3′ region of lncRNA 00312. The interbinding function of lncRNA 00312 and miR-34a-5p was also confirmed by luciferase report assay. The miR-34a-5p has been proved to have important biological functions in many tumors, including RCC [23, 26, 27]. In addition, we further demonstrated that miR-34a-5p is involved in the inhibitory role of lncRNA 00312 on invasion and proliferation of RCC. Furthermore, other miRNAs may be related to the inhibitory effect of lncRNA 00312 in RCC. miR-197-3p is the target for lncRNA 00312 inhibiting the invasive and migrating ability of the bladder cancer cells [28] and thyroid cancer cells [29]. miR-21 is the target for lncRNA 00312 inhibiting proliferation and metastasis of colorectal cancer cells [21]. We would study the relationship between lncRNA 00312 and other miRNAs later.

Previous studies also showed that argininosuccinate synthase 1 (ASS1) is a target of miR-34a-5p and can be negatively regulated by miR-34a-5p [23]. ASS1, an enzyme that catalyzes the synthesis of arginine succinic acid by citrulline and aspartic acid, catalyzes the reaction of citrulline with aspartic acid to produce arginine succinic acid [30]. Abnormally activated ASS1 gene plays an important role in tumorigenesis and development [31]. ASS1 and its pseudogene ASS1P3 were abnormally expressed in RCC.

To explore whether lncRNA 00312 inhibits the invasion and proliferation of RCC cells through ASS1, we cotransfected lncRNA 00312 overexpression vector and ASS1 siRNA vector into RCC cells. Our results showed that overexpression of lncRNA 00312 can increase the content of ASS1. And inhibition of ASS1 can significantly reverse the role of lncRNA 00312 in RCC. These data demonstrated that lncRNA 00312 inhibits the invasion and proliferation while promoting apoptosis of RCC cells; miR-34a-5p and ASS1 were involved in those physiological processes. We assumed that lncRNA 00312 could function as a ceRNA; when it binds to miR34a-5p, miR34a-5p may not be able to bind to ASS1 gene to downregulate the ASS1 expression, so lncRNA 00312 may exert antitumor capacity by normalizing ASS1 expression.

In conclusion, we conducted functional experiment and mechanism of lncRNA 00312 in RCC. lncRNA 00312 inhibited the proliferation and invasion while promoting apoptosis of RCC cells, which could be blocked by miR-34a-5p overexpression or ASS1 inhibition. Our study demonstrated that the lncRNA 00312/miR-34a-5p/ASS1 axis may play an important role in the progression of RCC, which provides new targets for clinical treatment.

## Figures and Tables

**Table 1:** Association between lncRNA 00312 expression and clinicopathological characteristics in 47 renal cell carcinoma patients.

Variable	lncRNA 00312 expression		*χ* ^2^/*t*	*P* value
	Low expression (*n* = 22)	High expression (*n* = 25)		
Age (years)	55.9 ± 12.1	56.5 ± 13.6	0.151^∗^	0.881
<60	12	14	0.010	0.920
≥60	10	11		
Gender				
Male	13	17	0.402	0.526
Female	9	8		
Pathological grade				
I–II	6	15	5.071	0.024
III–IV	16	10		
Lymph node involvement				
NO-N1	9	11	0.046	0.830
N2-NX	13	14		
Distant metastasis				
Absent	8	10	0.065	0.798
Present	14	15		
Tumor size				
T1-T2	8	17	4.704	0.030
T3-T4	14	8		
TNM stage				
I-II	7	16	4.850	0.028
III-IV	15	9		
ACR (mg/g)	35.04 ± 21.91	33.80 ± 25.09	-0.178^∗^	0.860
<30	14	16	0.078	0.781
≥30	8	9		
eGFR (mL/min/1.73m^2^)	90.1 ± 37.5	95.5 ± 39.0	0.484^∗^	0.631
≥60	16	18	0.074	0.786
<60	6	7		
Cr (*μ*mol/L)	92.1 ± 50.8	90.5 ± 51.2	-0.107^∗^	0.916
<82.1 (F)/97.0 (M)	18	20	0.046	0.831
≥82.1 (F)/97.0 (M)	4	5		
CysC (mg/L)	0.96 ± 0.54	0.94 ± 0.67	-0.115^∗^	0.909
<1.09	17	18	0.006	0.938
≥1.09	5	7		

Note: ^∗^Statistical *t* value by an independent samples *t*-test. The others were *χ*^2^ values by a chi-square test. eGFR: estimated glomerular filtration rate; ACR: urine albumin-creatinine ratio; CysC: cystatin C; Cr: creatinine; F: female; M: male.

**Table 2:** List for primers used for qRT-PCR.

Gene	Forward primer	Reverse primer
lncRNA 00312	5′-TCTGGCTGTTGTTGTGTTGGAGAAATA-3′	5′-GCTTATTGGCTTGGTTCGCT-3′
U1	5′-GGGAGATACCATGATCACGAAGGT-3′	5′-CCACAAATTATGCAGTCGAGTTTCCC-3′
GAPDH	5′-GGAGCGAGATCCCTCCAAAAT-3′	5′-GGCTGTTGTCATACTTCTCATGG-3′
miR-34a-5p	5′-GGGGTGGCAGTGTCTTAGC-3′	5′-CAGTGCGTGTCGTGGAGT-3′
U6	5′-CTCGCTTCGGCAGCACA-3′	5′-AACGCTTCACGAATTTGCGT-3′
ASS1	5′-TTGAAATTTGCTGAGCTGGTGTA-3′	5′-AGCCTGAGGGAATTGATGTTGAT-3′

## Data Availability

The data used to support the findings of this study are available from the corresponding author upon request.
